# Effect of COVID-19 lockdown on glycated haemoglobin testing and utilisation in KwaZulu-Natal, South Africa

**DOI:** 10.4102/ajlm.v13i1.2302

**Published:** 2024-05-06

**Authors:** Rucita Severaj, Verena Gounden

**Affiliations:** 1 Discipline of Chemical Pathology, Faculty of Laboratory Medicine, National Health Laboratory Service and University of KwaZulu-Natal, Durban, South Africa

**Keywords:** diabetes, glycated haemoglobin, HbA1c, glycaemic control, COVID-19, South Africa

## Abstract

**Background:**

Diabetic monitoring and treatment guidelines are easily accessible, but compliance is poor in KwaZulu-Natal. The coronavirus disease 2019 (COVID-19) pandemic had a devastating impact on diabetic healthcare, both directly and through public health interventions.

**Objective:**

This study aimed to close the gaps in knowledge concerning glycated haemoglobin (HbA1c) test utilisation and how this was affected by the COVID-19 lockdown in KwaZulu-Natal.

**Methods:**

We reviewed HbA1c test volumes and results from public health facilities in the 11 health districts in KwaZulu-Natal, South Africa. We compared testing trends and glycaemic control between two 10-month study periods before (March 2019 – December 2019) and during (March 2020 – December 2020) the COVID-19 pandemic.

**Results:**

The number of HbA1c tests performed decreased 6.1% during the pandemic period, with 173 760 HbA1c tests performed in 2019 and 163 236 HbA1c tests performed in 2020. There was a statistically significant increase in the average HbA1c level during the pandemic (mean HbA1c level in the pre-pandemic period: 70.5 mmol/mol [8.6%] versus mean HbA1c level in the pandemic period: 72.7 mmol/mol [8.8%]; *p*-value < 0.001). Of patients with suboptimal HbA1c results (83 421 in 2019, 83 259 in 2020), 11 656 (14.0%) in 2019 and 10 086 (12.1%) in 2020 had more than one HbA1c test performed during the study period.

**Conclusion:**

The COVID-19 pandemic impacted glycaemic monitoring in KwaZulu-Natal with lower HbA1c test volumes and worse glycaemic control seen during the pandemic. HbA1c testing practices are not in keeping with recommended guidelines.

**What this study adds:**

The study demonstrates that the COVID-19 pandemic impacted HbA1c utilisation in KwaZulu-Natal. Importantly, HbA1c testing practices in KwaZulu-Natal are not in keeping with Society for Endocrinology, Metabolism and Diabetes of South Africa guidelines regarding the monitoring of diabetic patients, and this requires more attention for future diabetic healthcare interventions.

## Introduction

Diabetes mellitus poses a major public health concern worldwide, especially in developing countries where diabetes prevalence is increasing at an alarming rate.^1^ In 2020, the International Diabetes Federation estimated that over 4.5 million adult South Africans were living with diabetes.^2^ Of greater concern is that an estimated 66.7% of diabetes cases in sub-Saharan Africa are undiagnosed, the highest of any other region in the world.^3^

KwaZulu-Natal is South Africa’s second most populous province, with an estimated population of 11.5 million (19% of the South African population) people.^4^ The burden of diabetes mellitus in KwaZulu-Natal is increasing, and poor glycaemic control is a major contributor to this.^5,6^ The Society for Endocrinology, Metabolism and Diabetes of South Africa’s 2017 guidelines define optimal glycaemic control as glycated haemoglobin (HbA1c) levels < 53 mmol/mol (< 7.0%), which is in keeping with global recommendations.^3^ Only 8.3% of diabetic patients attending a regional hospital in KwaZulu-Natal and 5.3% of diabetic patients managed at a central hospital in KwaZulu-Natal achieved optimal glycaemic control.^7,8^

HbA1c testing is the standard of care for the monitoring of treatment and glycaemic control in diabetic patients, both globally and within South Africa.^3,9^ HbA1c is preferred as it reflects the average plasma glucose over the previous 8 weeks to 12 weeks and can be performed at any time of the day, not requiring special preparations such as fasting.^10^ The Society for Endocrinology, Metabolism and Diabetes of South Africa’s 2017 guidelines recommend six-monthly HbA1c testing in patients with stable glycaemic control and three- to six-monthly HbA1c testing in those who have yet to achieve optimal control.^3^

Compliance with diabetic monitoring and treatment guidelines in public health institutions in KwaZulu-Natal is generally poor, as demonstrated by a study performed in a regional hospital in 2010 which found that monitoring and treatment guidelines were not followed in up to 80% of patients with poor glycaemic control,^11^ possibly due to lack of continuity of care and deficiencies of knowledge regarding recommended guidelines. This is problematic, because reduced HbA1c testing frequency is significantly associated with poor diabetes control,^12^ whilst adequate monitoring of diabetes patients is known to improve glycaemic control.^13^

The coronavirus disease 2019 (COVID-19) pandemic had a devastating effect on healthcare globally, both directly and indirectly. In addition to causing severe illness and resulting in the deaths of millions of people throughout the world, responses to the pandemic have had far-reaching consequences on the health and well-being of patients. These indirect effects occurred due to government responses to the pandemic, including lockdowns and the lifestyle changes associated with this, changed priorities of medical and surgical procedures, cessation of screening programmes, altering medical facilities to COVID-19 centres, public fear of seeking healthcare, and reduced referrals.^14^ A World Health Organization survey found that disruption to healthcare service, especially primary healthcare, was greatest among lower-income countries where overburdened healthcare systems, high rates of both communicable and non-communicable diseases, and poor socioeconomic determinants allowed the pandemic to exacerbate pre-existing vulnerabilities.^15,16^

In South Africa, lockdown measures were implemented based on ‘alert’ levels, with the most restrictive measures seen in March 2020 – August 2020. These measures directly affected ambulatory clinic visitations and the management of many chronic diseases. Health services relating to reproductive health, maternal and child health, HIV and tuberculosis, and diabetes and hypertension were the hardest hit because of decreased outpatient visits during this period.^17,18^

Several studies have investigated the effects of the COVID-19 lockdown on HbA1c testing and glycaemic control, with highly variable results. Studies performed in India and Turkey reported up to 70% reduction in HbA1c testing volumes for outpatients during the lockdown, with an associated increase in HbA1c levels.^19,20,21^ Others studies performed in Denmark and Italy found no significant difference in glycaemic control during the lockdown periods in their respective countries,^22,23^ and other studies from the United Kingdom and Spain even demonstrated improved glycaemic control during lockdown periods.^24,25^ Improved control was often seen in developed, higher-income countries where telemedicine, home HbA1c monitoring, and mobile phlebotomy units are available and easily accessible.^26,27^

Only a few studies have investigated HbA1c utilisation in diabetic patients managed at public health facilities in KwaZulu-Natal and how this relates to glycaemic control and established practice guidelines. Most of the published studies were performed at an institutional level, with little information at the provincial or district levels. In addition, no local studies have investigated the effect of the COVID-19 lockdown on HbA1c testing and glycaemic control. This information is essential for public health planning and policy development, especially in light of increased demands on smaller healthcare budgets. This study thus attempts to close the gaps in knowledge concerning HbA1c testing and glycaemic control, and how these parameters have been affected by the COVID-19 lockdown in KwaZulu-Natal.

## Methods

### Ethical considerations

Ethics approval for this study was obtained from the University of KwaZulu-Natal, Biomedical Research Ethics Committee (BREC/00004109/2022). Approval to conduct research was also obtained from the KwaZulu-Natal Department of Health (KZ_202206_024). The requirement for patient consent was waived by the ethics committee. Data were collected on a password-protected computer accessible by only the primary investigator. Patients were identified by unique patient identification numbers which did not contain identifiable patient data and their identities were not revealed.

### Data collection

HbA1c data from all National Health Laboratory Service laboratories in the province of KwaZulu Natal were extracted from the National Health Laboratory Service Central Data Warehouse. The 11 health districts included were Amajuba, eThekwini, Harry Gwala, Ilembe, King Cetshayo, Ugu, uMgungundlovu, Umkhanyakude, Umzinyathi, Uthukela and Zululand. Data from two 10-month study periods were included. Period 1 (01 March 2019 to 31 December 2019) was the pre-pandemic period, and Period 2 (01 March 2020 to 31 December 2020) was during the pandemic. Two methods were used to analyse HbA1c across all laboratories: ion exchange high performance liquid chromatography and immunoassay. All methods were National Glycohemoglobin Standardization Program traceable, and there was harmonisation between methods allowing for comparison of results from different laboratories. Variables extracted included demographic information, specifically the age and sex of the patient, district and facility name, clinical diagnosis, date reviewed, HbA1c result (percentage) and HbA1c text which provided information regarding less than (<) or greater than (>) HbA1c results and HbA1c variants. All patients with at least one HbA1c result within the study periods were included. Results with a lower than detectable value, unfeasible results, and HbA1c tests where a result was not provided, such as in the case of interfering haemoglobin variants, were excluded. Based on their infrastructure and development, the eThekwini, Ilembe, uMgungundlovu and Ugu districts were classified as urban, and the other districts were classified as rural. ‘Strict’ lockdown levels were defined as all alert levels greater than alert level 1.

### Data analysis

Data analysis was carried out using Microsoft Excel 2016 (Microsoft Corporation, Redmond, Washington, United States). We determined the cumulative total number of HbA1c tests conducted during each study period, both as an overall aggregate and separately for each of the 11 health districts in KwaZulu-Natal. We determined the impact of the pandemic by comparing the testing volumes, average HbA1c levels, and the percentage of abnormal HbA1c results (defined as HbA1c levels > 53 mmol/mol [> 7.0%]) in both period 1 and period 2. We also plotted the number of HbA1c test results that fell within different HbA1c ranges, and compared pre-pandemic results with results from during the pandemic. Statistical significance was determined with the use of a Student t-test for the average HbA1c level, and a Chi squared test for the percentage of abnormal HbA1c results. A *p*-value of < 0.05 was considered significant.

To gauge whether HbA1c utilisation was in keeping with local guidelines, we looked at the total number of patients who had repeat HbA1c tests performed within the study periods. We also looked at the number of patients who had a single HbA1c test that was performed within the first 4 months (March – June) of a study period. These data were extracted using unique patient identification numbers. We also looked particularly at the number of patients with suboptimal HbA1c levels, which we defined as an HbA1c > 58.5 mmol/mol (> 7.5%), who had repeat HbA1c tests performed within the study periods.

## Results

**FIGURE 1:**
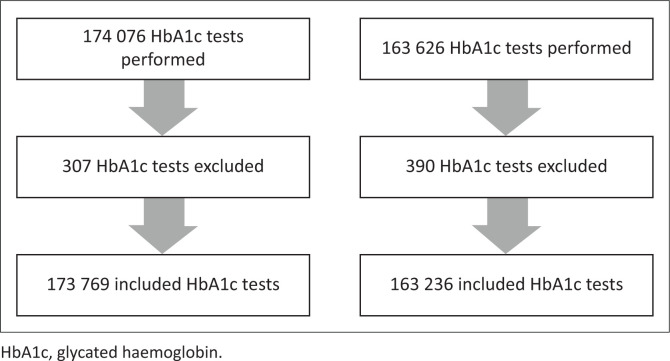
Inclusion criteria applied to glycated haemoglobin (HbA1c) test results of all patients attending public health facilities in KwaZulu-Natal, South Africa, 01 March 2019 – 31 December 2019 and 01 March 2020 – 31 December 2020.

A total of 174 076 HbA1c tests were performed between March 2019 and December 2019 compared to 163 626 tests performed during the same period in 2020 (Figure 1). Of these, 307 tests conducted in 2019 and 390 conducted in 2020 were excluded. Excluded results included 109 lower than detectable results in 2019 and 209 in 2020, 198 interfering HbA1c variants in 2019 and 176 in 2020, and 5 unfeasible results in 2019 and 9 in 2020. Unfeasible HbA1c results included results of 0.0% and 1065.0%. In total, 173 769 HbA1c test results for 2019 and 163 236 results for 2020 were included in this study.

Approximately two-thirds (67%) of the results were from female patients, and over half of the results (55%) were from patients aged between 50 years and 70 years (Table 1). Notably, the demographic distribution was similar in the pre-pandemic and pandemic periods.

**TABLE 1:** Demographic data of patients attending public health facilities in KwaZulu-Natal, South Africa, 01 March 2019 – 31 December 2019 and 01 March 2020 – 31 December 2020.

Year	Month	Male		Female		Unknown		Age range (years)																	
								< 18		18–30		31–40		41–50		51–60		61–70		71–80		> 80		Unknown	
		*n*	%	*n*	%	*n*	%	*n*	%	*n*	%	*n*	%	*n*	%	*n*	%	*n*	%	*n*	%	*n*	%	*n*	%
2019	March	5228	29	12 266	68	590	3	238	1	620	3	1233	7	2525	14	5026	28	4827	27	2546	14	664	4	405	2
	April	4738	29	10 992	67	555	3	243	1	575	4	1111	7	2356	14	4427	27	4406	27	2177	13	587	4	403	2
	May	5355	30	11 658	66	548	3	251	1	686	4	1202	7	2525	14	4825	27	4668	27	2288	13	672	4	444	3
	June	4801	30	10 609	67	509	3	237	1	546	3	1083	7	2186	14	4417	28	4246	27	2198	14	640	4	366	2
	July	5813	29	13 261	67	649	3	250	1	677	3	1281	6	2729	14	5412	27	5477	28	2706	14	729	4	462	2
	August	5898	30	13 384	67	648	3	316	2	710	4	1245	6	2737	14	5557	28	5410	27	2731	14	802	4	422	2
	September	4962	29	11 436	67	595	4	219	1	605	4	1107	7	2389	14	4680	28	4624	27	2308	14	682	4	379	2
	October	6336	31	13 702	66	670	3	307	1	714	3	1462	7	2947	14	5754	28	5566	27	2810	14	785	4	363	2
	November	5288	31	11 020	65	560	3	234	1	667	4	1215	7	2326	14	4617	27	4626	27	2139	13	695	4	349	2
	December	3650	31	7645	65	403	3	179	2	534	5	861	7	1616	14	3065	26	3067	26	1677	14	489	4	210	2
2020	March	5191	30	11 605	67	561	3	212	1	664	4	1168	7	2333	13	4716	27	4839	28	2444	14	652	4	329	2
	April	3662	31	7641	66	354	3	119	1	440	4	856	7	1661	14	3277	28	3172	27	1536	13	426	4	170	1
	May	4341	30	9487	67	431	3	189	1	550	4	1069	7	2086	15	4078	29	3778	26	1824	13	480	3	205	1
	June	5305	31	11 000	65	541	3	248	1	639	4	1275	8	2450	15	4638	28	4415	26	2315	14	594	4	272	2
	July	5232	31	11 007	66	490	3	193	1	640	4	1293	8	2446	15	4655	28	4429	26	2131	13	570	3	372	2
	August	4919	31	10 448	66	472	3	161	1	631	4	1198	8	2350	15	4493	28	4151	26	2012	13	497	3	346	2
	September	5499	31	11 918	66	541	3	188	1	716	4	1348	8	2684	15	4984	28	4742	26	2284	13	618	3	394	2
	October	6018	29	13 956	68	611	3	262	1	771	4	1586	8	2915	14	5625	27	5479	27	2787	14	749	4	411	2
	November	5270	29	12 184	68	536	3	236	1	776	4	1436	8	2636	15	4762	26	4891	27	2297	13	643	4	313	2
	December	4274	30	9242	66	500	4	189	1	605	4	1136	8	2076	15	3675	26	3713	26	1788	13	508	4	326	2

A reduction in HbA1c tests performed (6.1%) was seen in the pandemic period compared to the pre-pandemic period (Table 2). Most of the HbA1c tests (50% in 2019 and 43% in 2020) came from the major metropolitan area of Ethekwini, followed by uMgungundlovu and Ilembe. These three health districts were also the only ones to have had fewer HbA1c tests performed during the pandemic compared to the pre-pandemic period. The other eight health districts had more HbA1c tests in 2020. A significant increase in average HbA1c of 2.2 mmol/mol (0.2%) was seen during the pandemic. Nine of the 11 health districts demonstrated a higher average HbA1c in 2020 compared to 2019. Harry Gwala district showed a decrease in average HbA1c, but this was minimal (0.2%).

**TABLE 2:** Number of glycated haemoglobin (HbA1c) tests and average HbA1c, and percent difference† of all patients attending public health facilities in KwaZulu-Natal, South Africa, 01 March 2019 – 31 December 2019 and 01 March 2020 – 31 December 2020.

District	HbA1c tests performed			Average HbA1c (mmol/mol)						
	2019 (*n*)	2020 (*n*)	Difference (%)	2019		2020		Difference		*p*
				mmol/mol	%	mmol/mol	%	mmol/mol	%	
Total	173 760	163 236	−6.1	70.5	8.6	72.7	8.8	2.2	0.2	< 0.001
Amajuba	5724	6132	7.1	78.1	9.3	79.2	9.4	1.1	0.1	0.037
eThekwini	86 203	70 990	−17.6	67.2	8.3	69.4	8.5	2.2	0.2	< 0.001
Harry Gwala	2805	3697	31.8	85.8	10.0	83.6	9.8	−2.2	−0.2	0.030
Ilembe	15 665	13 349	−14.8	73.8	8.9	76.0	9.1	2.2	0.2	< 0.001
King Cetshwayo	11 649	12 765	9.6	76.0	9.1	78.1	9.3	2.1	0.2	< 0.001
Ugu	9968	11 382	14.2	70.5	8.6	73.8	8.9	3.3	0.3	< 0.001
uMgungundlovu	19 012	19 220	1.1	66.1	8.2	66.1	8.2	0.0	0.0	0.824
Umkhanyakude	4982	5411	8.6	76.0	9.1	78.1	9.3	2.1	0.2	0.004
Umzinyathi	2702	3605	33.4	79.2	9.4	82.5	9.7	3.3	0.3	< 0.001
Uthukela	6177	6486	5.0	70.5	8.6	71.6	8.7	1.1	0.1	0.001
Zululand	8873	10 199	14.9	81.4	9.6	82.5	9.7	1.1	0.1	0.012

Note: Student t-test used to determine statistical significance. Absolute Difference = 2020 value – 2019 value.

HbA1c, glycated haemoglobin.

† , Percent difference = (2019 value – 2020 value)/2019 value.

**FIGURE 2:**
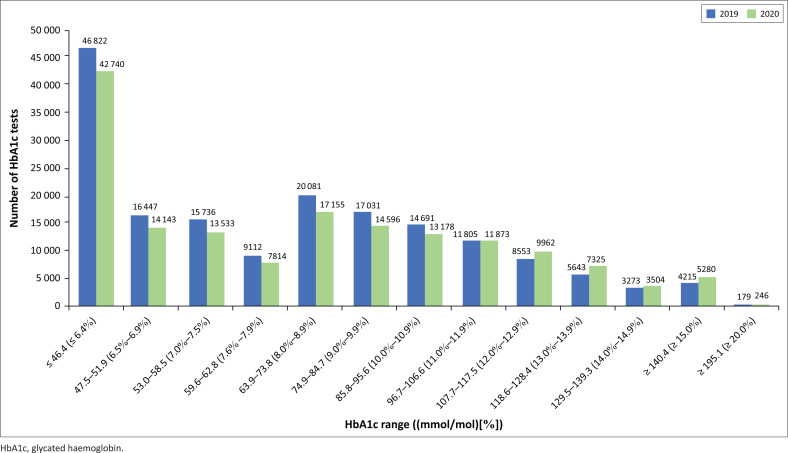
Distribution of glycated haemoglobin (HbA1c) results according to HbA1c range of all patients attending public health facilities in KwaZulu-Natal, South Africa, 01 March 2019 – 31 December 2019 and 01 March 2020 – 31 December 2020.

The distribution of HbA1c results by HbA1c value range demonstrated fewer HbA1c results in the low ranges (below 85.8 mmol/mol; 10%) in 2020 compared to the same period in 2019 (Figure 2). Conversely, there were more HbA1c results in the high ranges (especially above 96.7 mmol/mol; 11%) in 2020 compared to 2019.

**FIGURE 3:**
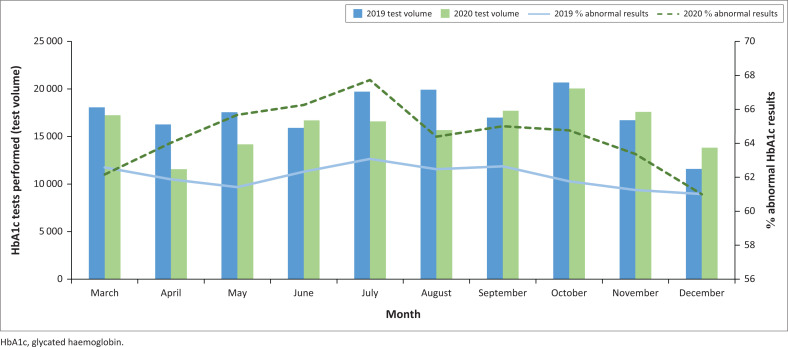
Volume of glycated haemoglobin (HbA1c) tests and percentage of abnormal results per month for all patients attending public health facilities in KwaZulu-Natal, South Africa, 01 March 2019 – 31 December 2019 and 01 March 2020 – 31 December 2020. Abnormal was defined as HbA1c levels > 53 mmol/mol (> 7.0%).

There were much lower test volumes in the first few months of the pandemic (March, April, May, July and August 2020) compared to the same period in 2019 (Figure 3). This trend was reversed during the pandemic, with higher test volumes recorded in September 2020, November 2020, and December 2020 compared to the same months in 2019. The HbA1c test volume per month for each district followed a similar pattern as described for the total volumes (Online Supplementary Figure 1). A significant increase in the percentage of abnormal HbA1c results is seen during the pandemic for the months April 2020 – November 2020 (Table 3). The highest difference in the percentage of abnormal HbA1c results were seen in May 2020 – July 2020, the period following the strictest lockdown levels, alert levels 5 and 4. The percentage of abnormal results recorded per month in each district did not show much variation between the pre-pandemic and pandemic periods, (Online Supplementary Table 1). Five of the health districts had more abnormal results in 2020 than 2019 (Online Supplementary Figure 2).

**TABLE 3:** Volume of glycated haemoglobin (HbA1c) tests and percentage of abnormal results per month for all patients attending public health facilities in KwaZulu-Natal, South Africa, 01 March 2019 – 31 December 2019 and 01 March 2020 – 31 December 2020.

Month	2019			2020			Difference in abnormal results (%)	*p*
	Total HbA1c tests (*n*)	Total abnormal HbA1c tests (*n*)	Abnormal HbA1c tests (%)	Total HbA1c tests (*n*)	Total abnormal HbA1c tests (*n*)	Abnormal HbA1c tests (%)		
March	18 069	11 307	62.58	17 237	10 715	62.16	−0.41	< 0.001
April	16 268	10 066	61.88	11 568	7407	64.03	2.15	< 0.001
May	17 554	10 782	61.42	14 174	9309	65.68	4.25	< 0.001
June	15 911	9917	62.33	16 692	11 060	66.26	3.93	< 0.001
July	19 716	12 437	63.08	16 592	11 237	67.73	4.64	< 0.001
August	19 923	12 447	62.48	15 677	10 095	64.39	1.92	< 0.001
September	16 989	10 643	62.65	17 704	11 509	65.01	2.36	< 0.001
October	20 678	12 771	61.76	20 045	12 981	64.76	3.00	< 0.001
November	16 717	10 239	61.25	17 591	11 147	63.37	2.12	< 0.001
December	11 593	7073	61.01	13 825	8433	61.00	−0.01	0.983

**Total**	**173 418**	**107 682**	**62.09**	**161 105**	**103 893**	**64.49**	**2.39**	**< 0.001**

Note: Statistical significance determined using Pearson’s Chi squared test. Percentage abnormal HbA1c tests = (Total number of abnormal HbA1c tests/total number of HbA1c tests)*100. Difference in abnormal results = 2020 percentage abnormal HbA1c tests – 2019 percentage abnormal HbA1c tests. Abnormal was defined as HbA1c levels > 53 mmol/mol (> 7.0%).

HbA1c, glycated haemoglobin.

**FIGURE 4:**
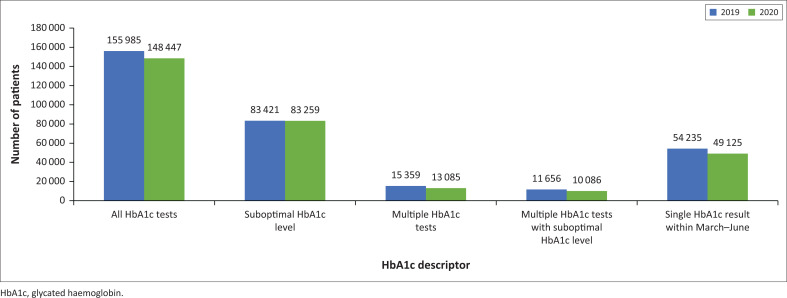
Glycated haemoglobin (HbA1c) testing frequency among patients attending public health facilities in KwaZulu-Natal, South Africa, 01 March 2019 – 31 December 2019 and 01 March 2020 – 31 December 2020. Suboptimal HbA1c level was defined as > 58.5 mmol mol (> 7.5%).

HbA1c tests were conducted for 155 985 patients in 2019 and 148 447 patients in 2020 (Figure 4). Of these, only 15 359 (9.8%) patients in 2019 and 13 085 (8.8%) in 2020 had more than one HbA1c test performed within the 10-month study period. Suboptimal HbA1c levels were reported for 83 421 (53.5%) patients in 2019 and 83 259 (56.0%) patients in 2020. Of those patients, only 11 656 (14.0%) in 2019 and 10 086 (12.1%) in 2020 had more than one HbA1c test performed. There were 54 235 patients in 2019 and 49 125 patients in 2020 who only had a single HbA1c test performed within the study period and this test was within the first 4 months of the study period. Two HbA1c tests per patient (13 358/15 359 patients in 2019 and 11 582/13 085 patients in 2020) was the most common test frequency for patients who had multiple HbA1c tests conducted (Online Supplementary Table 2).

## Discussion

This study demonstrated that the COVID-19 pandemic did impact HbA1c utilisation in patients attending public health facilities. Lower HbA1c test volumes were recorded during the pandemic in 2020 than during the same period pre-pandemic in 2019. A significant increase in the average HbA1c level was seen during the pandemic as well as significantly higher abnormal HbA1c results, particularly during periods of strict lockdown, when HbA1c test volumes were at their lowest. It was also demonstrated that pre-pandemic and pandemic HbA1c testing practices were not in keeping with established International and National guidelines regarding the monitoring of glycaemic control in diabetic patients. Fewer HbA1c tests are being performed on sub-optimally controlled diabetic patients than should be as per the guidelines.

Several studies have been conducted globally, particularly in high-income countries, to investigate the impact of the COVID-19 pandemic on diabetes care. Studies performed in the United Kingdom in 2021 and Canada in 2020, found that monthly HbA1c requests during the pandemic dropped to 7.9% – 18.1% of pre-pandemic volumes and patients had a 28% lower probability of undergoing an HbA1c test during the pandemic.^28,29^ Studies conducted among North American patients in 2020 and 2021, respectively, found that HbA1c testing frequency during the pandemic reduced by as much as 66%,^30^ and was at 75% – 85% of the rate of prior years’.^31^ The findings of our study mirrored these, where a reduction of HbA1c test volumes was seen during the pandemic compared to the same period in 2019. However, we found this reduction to be more subtle, at 6.1%, compared to reductions of between 50% and 70% reported by these studies from higher-income settings.^28,29,30,31^ Interestingly, reduced HbA1c test volumes were only noted in two of the 11 KwaZulu-Natal health districts – eThekwini, the metropolitan (−17.5%) and Ilembe (−14.8%). These districts have a relatively higher level of urban development in terms of setting and infrastructure. In the other more rural districts, increases in HbA1c test volumes by as much as 33.4% (Umzinyathi) were reported during the pandemic. Although such increases have not been reported in other studies around the world, the paucity of studies performed in rural populations is a likely contributing factor. Another consideration is that our study was performed using data from the public health service in KwaZulu-Natal, where remote forms of healthcare such as telemedicine were not available to the study population. The inability to consult remotely with healthcare practitioners meant that diabetic patients in KwaZulu-Natal continued with only in-person health consults. For the majority of patients from these rural and semi-urban areas, there would have been no alternatives to access healthcare and medication without face-to-face visits to the primary care clinics or hospitals. Thus, the frequency of patient visits would not have been significantly decreased by the pandemic in these districts. Whilst awareness by clinicians regarding the poorer outcomes of diabetic individuals infected by COVID-19 may have increased the frequency of HbA1c testing, this is not substantiated by the testing volumes across all the districts.

The most notable decline in HbA1c test volume was recorded between April 2020 and August 2020, during which the strictest levels of lockdown were in place in South Africa. HbA1c test volumes recovered in September 2020, and exceeded 2019 volumes in November 2020 and December 2020. This finding concurs with studies conducted in the United States, Canada and Spain between 2020 and 2021.^19,31,32^

Despite the consistent evidence of decreased HbA1c testing during the pandemic, studies have demonstrated variable effects of this on glycaemic control. Studies conducted in China in 2022 and Taiwan in 2021 found no significant difference in HbA1c results between the pandemic and pre-pandemic periods.^33,34^ Remarkably, a study conducted in Spain in 2021 instead reported improved glycaemic control during the pandemic, and this was supported by two systematic reviews and meta-analyses performed in the United Kingdom and Germany in 2021.^35,36,37^ In contrast to the findings of these studies, we found a significant increase in the average HbA1c result during the pandemic of 2.2 mmol/mol (0.2%) overall and in nine of the districts, indicating poorer glycaemic control during the pandemic where decreased numbers of HbA1c tests were being conducted. Notably, the Harry Gwala district, which had an increase of 31.8% in HbA1c tests performed during the pandemic, also demonstrated a significantly improved pandemic average HbA1c result. This study also found that there were more patients with high-range HbA1c levels during the pandemic than before the pandemic, an indication of poorer glycaemic control in sub-optimally controlled patients. This may suggest lack of prioritisation of poorly controlled diabetic patients during the pandemic similar to the findings of Holland et al. from the United Kingdom in 2021.^28^

Considerably fewer studies have investigated the effect of the pandemic on glycaemic control in diabetic patients in low- and middle-income settings. Most of those performed were concerned with poor health outcomes in diabetic patients with COVID-19 and had small sample sizes and cohorts of admitted patients during the pandemic.^38,39,40,41^ A study performed in a lower-middle income setting in South Asia in 2020 demonstrated a decrease in mean HbA1c level by 0.3% during the pandemic and found improved HbA1c results in 37.6% of participants.^42^ A South African study performed in Gauteng province in 2021, found similar findings to our study, where they showed decreased HbA1c testing volumes during the COVID-19 lockdown periods and, while it reported variability in HbA1c results and glycaemic control between these pre-pandemic and pandemic periods, the significance of this was not determined.^40^ A smaller study which included only 28 participants, conducted in 2020 in the Western Cape, South Africa reported a sharp decline in HbA1c test requests compared to the same period the year before; however, they did not quantify this decline or explore how this related to glycaemic control.^41^ While our study demonstrated similar decreased HbA1c test volumes during the pandemic as described by these three studies, the finding of a significantly increased average HbA1c result between pre-pandemic and pandemic periods is novel in this middle-income setting.

Our study also demonstrated that significantly more abnormal HbA1c results were recorded from April 2020 to August 2020, during the stricter lockdown periods, compared to pre-pandemic values from the same period in 2019. This relates to the same period where the most notable decreases in HbA1c testing are seen. This increase in abnormal HbA1c results during these ‘hard’ lockdown periods may be related to both the indirect effects of the pandemic, such as the reduced access to healthcare, and reduced glycaemic monitoring. It is also possibly a consequence of the direct effects of COVID-19 on patients living with diabetes; however, without access to comorbidity data, we were unable to test this hypothesis.

The latest guidelines published in the Journal of Endocrinology, Metabolism and Diabetes of South Africa in 2017 recommend 6-monthly HbA1c testing in patients with stable control and 3-monthly testing in those with suboptimal glycaemic control.^3^ The proportion of patients who had multiple HbA1c tests performed was alarmingly low in both the pre-pandemic period (9.8%) and the pandemic period (8.8%). Notably, 54 235 patients (35.0% of all patients) in 2019 and 49 125 patients (33.0% of all patients) in 2020 had an HbA1c performed within the first 4 months of their respective study periods, yet did not have a repeat HbA1c performed. This is a clear indication that guidelines are not being followed appropriately. Of the small number of patients who had multiple HbA1c tests performed, the overwhelming majority had two HbA1c tests done, which may be appropriate in well-controlled patients. However, considering that over 50% of patients (53.5% in 2019 and 56.0% in 2020) included in each study period had suboptimal HbA1c levels (defined as HbA1c > 7.5%), this low frequency of HbA1c testing is more noteworthy. The low rate of follow-up HbA1c tests being performed overall is of great concern and is a likely contributor to the high burden of diabetes morbidity and mortality experienced in South Africa. This finding highlights a crucial gap in the management of diabetic patients in KwaZulu-Natal and identifies an area of focus for future interventions to improve diabetic care in the province and country.

The strength of our study lies in its uniqueness, as studies of this magnitude investigating HbA1c utilisation in low- and middle-income settings are very few. No such study has also included results from two 10-month periods. The 10-month pandemic period chosen for this study included the period when South Africa implemented strict lockdown measures as well as some months after the lockdown was lifted, thus allowing us to review the effect of the lockdown on HbA1c testing practices. Additionally, the study included both paediatric and adult populations in whom HbA1c testing is equally important. This study also analysed the data per health district in the province, thus providing insights into HbA1c utilisation in each province during the pandemic and allowing us to discriminate between urban and rural populations.

### Limitations

One limitation of this study is that we considered a single indicator of glycaemic control, HbA1c. HbA1c is accepted as the preferred test for the monitoring of glycaemic control in diabetic patients and because we were looking at a large number of results over 20 months, we chose to analyse a single and robust indicator. Another limitation of the study is the lack of clinical information available to us due to the method of data collection used. As such we were unable to differentiate HbA1c tests performed for diagnosis or monitoring.

### Conclusion

HbA1c utilisation in KwaZulu-Natal health districts decreased during the COVID-19 pandemic when public health interventions restricted access to routine medical care. This decrease, however, was much lower than seen in higher-income settings, where remote healthcare services such as telemedicine are accessible. The decreased HbA1c testing during the pandemic was a possible contributor to significant changes in glycaemic control seen. Most concerning, HbA1c utilisation in general is not in keeping with established diabetic monitoring guidelines.
